# Coupled Mechanisms of Precipitation and Atomization in Burning Nanofluid Fuel Droplets

**DOI:** 10.1038/srep15008

**Published:** 2015-10-08

**Authors:** Ankur Miglani, Saptarshi Basu

**Affiliations:** 1 Department of Mechanical Engineering, Indian Institute of Science, Bangalore, 560012, India

## Abstract

Understanding the combustion characteristics of fuel droplets laden with energetic nanoparticles (NP) is pivotal for lowering ignition delay, reducing pollutant emissions and increasing the combustion efficiency in next generation combustors. In this study, first we elucidate the feedback coupling between two key interacting mechanisms, namely, secondary atomization and particle agglomeration; that govern the effective mass fraction of NPs within the droplet. Second, we show how the initial NP concentration modulates their relative dominance leading to a master-slave configuration. Secondary atomization of novel nanofuels is a crucial process since it enables an effective transport of dispersed NPs to the flame (a pre-requisite condition for NPs to burn). Contrarily, NP agglomeration at the droplet surface leads to shell formation thereby retaining NPs inside the droplet. In particular, we show that at dense concentrations shell formation (master process) dominates over secondary atomization (slave) while at dilute particle loading it is the high frequency bubble ejections (master) that disrupt shell formation (slave) through its rupture and continuous outflux of NPs. This results in distinct combustion residues at dilute and dense concentrations, thereby providing a method of manufacturing flame synthesized microstructures with distinct morphologies.

The significance of secondary atomization in burning nanofuel droplets lies in its ability to transport the dispersed nanophase additives from the droplet to the flame zone and distribute them homogenously along with the fuel charge^1^,^2^,^3^,^4^,^5^,^6^,^7^,^8^. This is crucial in automotive diesel fuels where colloidal dispersions of nanocatalysts (having increased density of surface functionalities) can be used to reduce ignition delay and enhance combustion characteristics by catalyzing the reactions in the gas-phase reaction zone^9^,^10^. In colloidal droplets containing nanostructured ignition agents, secondary break-up can play a key role in achieving *distributed ignition* which markedly improves the combustion efficiency compared to *single-point ignition*^11^. Secondary break-up or disruption of burning nanofuel droplets exhibits a bi-modal behavior. First, the droplet may fragment catastrophically, thereby, releasing the suspended nanoparticles (NP) during a single microexplosion event. Second, the droplet may undergo *continuous atomization* and transport NPs continuously across the droplet surface along with the carrier daughter droplets (DD) resulting from multiple ejections^6^,^7^,^8^. This results in integrated burning i.e. NPs and base fuel burn simultaneously. A typical ejection event features a sequential process of bubble collapse (i.e. gas escape), ligament inception/growth and finally ligament necking and fracture to form DDs^6^,^8^. At the droplet scale, these continuous ejections induce bulk motion in the droplet (i.e. shifting of centroid) and corrugations on the droplet surface. This provides an enhanced surface area for fuel vaporization. Previous studies^7^,^8^ have shown that the latter mode of atomization is particularly attractive since it offers the ease of control both by excitation through external stimuli (preferential acoustic perturbations) and by varying functional properties (i.e. initial NP loading rate (PLR)). These studies focused on individual ejection modes/localized atomization events and characterized them based on their intensity (i.e. their droplet deformation inducing potential). Ejection intensity was defined based on two droplet deformation indices: (1) ejection impact parameter *α*_*local,max*_ (*t*) and (2) droplet void fraction *ξ*(*t*). Physical interpretation of these parameters and the classification of ejection modes^6^,^8^ based on these parameters is detailed in references^6^,^7^,^8^. In this work, however, we explain physically how the interplay between two key mechanisms affects the global atomization behavior of burning nanofuel droplets i.e. the *frequency of ejections f*(*t*) and the *shell formation by NP aggregation*. A dynamic feedback loop (involving these coupled mechanisms) that leads to distinct secondary break-up behavior at dilute and dense PLRs is illustrated in Fig. 1.

## Results

Scatter plot of Fig. 2 illustrates the spatial shifting of the *geometric droplet centroid* 
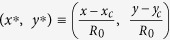
 and the area swept due to this centroid motion as a function of the PLR. Here (*x*, *y*) represents the instantaneous co-ordinates of the droplet centroid, 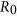 is the initial droplet radius (mm) and (*x*_*c*_, *y*_*c*_) is the correction term that accounts for the upward shift in the centroid due to reduction in the droplet diameter. Particularly, (*x*_*c*_, *y*_*c*_) is the centroid of an *assumed spherical droplet* that has same initial diameter (*D*_*0*_ = *2R*_*0*_) as the *actual droplet* and also shrinks isotropically at the same rate as the actual droplet.

Particularly, the total area swept by centroid motion during the droplet lifetime is an order higher at dilute PLRs compared to dense PLRs (*A* is confined to a small region surrounding the initial centroid (*x*_*0*_, *y*_0_)). On a time-averaged basis, the centroid motion over the entire droplet lifetime can be characterized by two global parameters: (1) the time-mean *relative area coverage* 
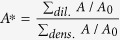
 which is ~6 times and (2) centroid position calculated based on the *weighted area* (swept area) *average* 
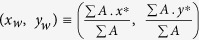
. Here, the *swept area A* for a particular concentration is defined as the total area covered due to centroid shifting and quantifies the bulk motion of the droplet induced by ejection events and 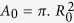 is the initial projected area of the droplet. In essence, 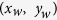 is a locator of the time-mean position of the centroid and measures the extent by which the centroid is constantly off-set from its initial position during entire droplet lifecycle (on an average) (Fig. 2). Clearly, at dense PLRs 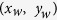 resides very close to the initial position i.e. 
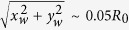
, thus, indicating that the centroid motion is rather stagnated at dense PLRs. Contrarily, at dilute PLRs, the weighted area averaged centroid is displaced substantially i.e. 
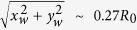
. Therefore, the swept area *A* can be used as one of the parameters to demarcate whether a given nanofuel droplet belongs to a family of droplets with dilute or dense initial PLRs.

**Figure 1:**
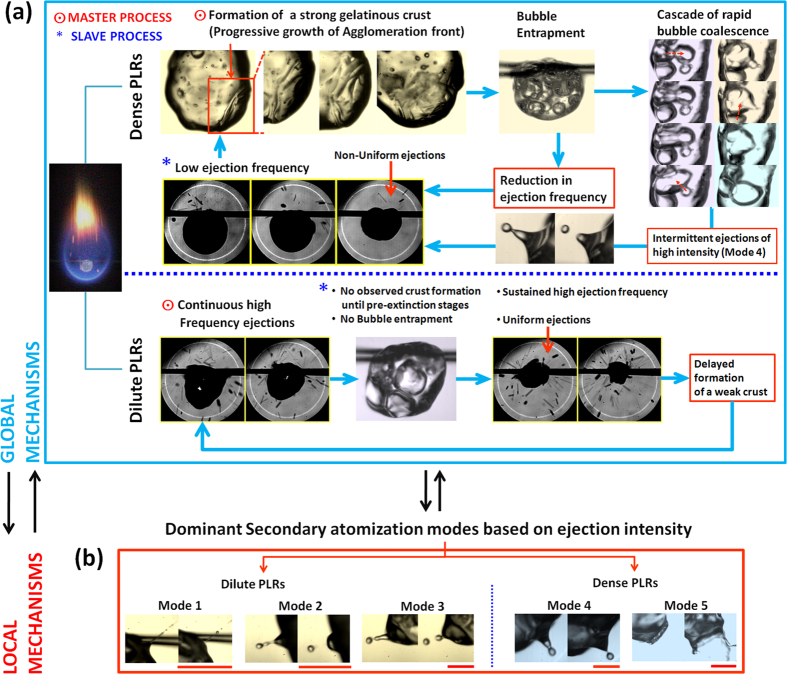
Interplay between global and local mechanisms that govern the overall secondary break-up behavior of a burning nanofluid fuel droplet. **(a)** Physical pathway involving coupled but competing *global mechanisms* of shell formation (through particle agglomeration) and frequency of ejections. Interplay between these two mechanisms is characterized by a master-slave system where the relative dominance of each is reversed as the PLR changes from initially dilute to initially dense. **(b)** High resolution (1024 × 1024), high speed images (15000 fps) of dominant atomization modes (adapted with permission from [8]). These *localized ejections* are characterized in the increasing order of intensity as: **Mode 1**: High kinetic energy Pin/ Needle-type ligament ejections with multiple point fracture (*α*_*local*_ ≪ 0.01); *ξ* ≈ 0, **Mode 2**: Needle ejection with both tip-base break-up (*α*_*local*_ ≪ 0.1), *ξ* ≈ 0, **Mode 3**: low momentum needle-type ligaments with only tip break-up (0.1 < *α*_*local*_ < 0.3); 0.1 < *ξ* < 0.2, **Mode 4**: low momentum, thick ligaments (*α*_*local*_ > 0.3); *ξ* > 0.3 and **Mode 5**: localized catastrophic fragmentation with multiple ligament formation (*α*_*local*_ → 1); *ξ* → 1. First three modes (i.e. Mode 1, 2 and 3) are low intensity ejections and dominant at dilute PLRs while latter two modes (Mode 4 and 5) are high intensity ejections and dominant at dense PLRs. All scale bars equal 1 mm.

**Figure 2:**
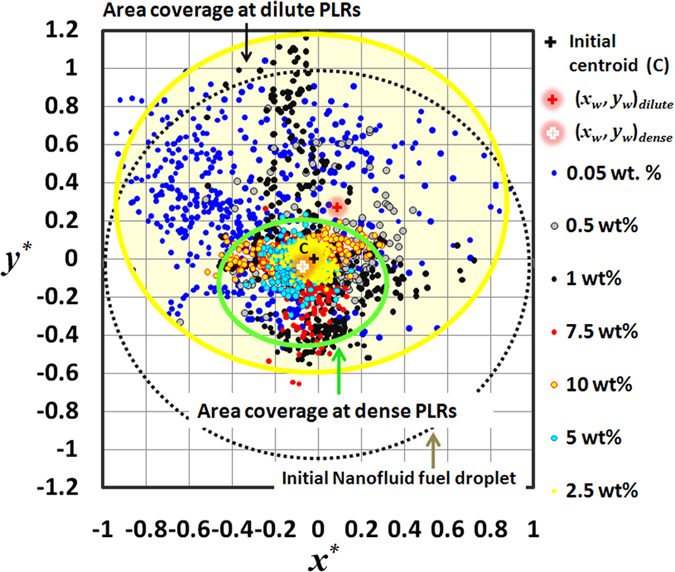
Spatial shifting of the instantaneous droplet centroid 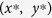 as a function of the initial NP loading rate. Time-mean position of the weighted area averaged centroid 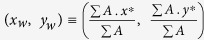 is marked for both dilute and dense PLRs.

**Figure 3:**
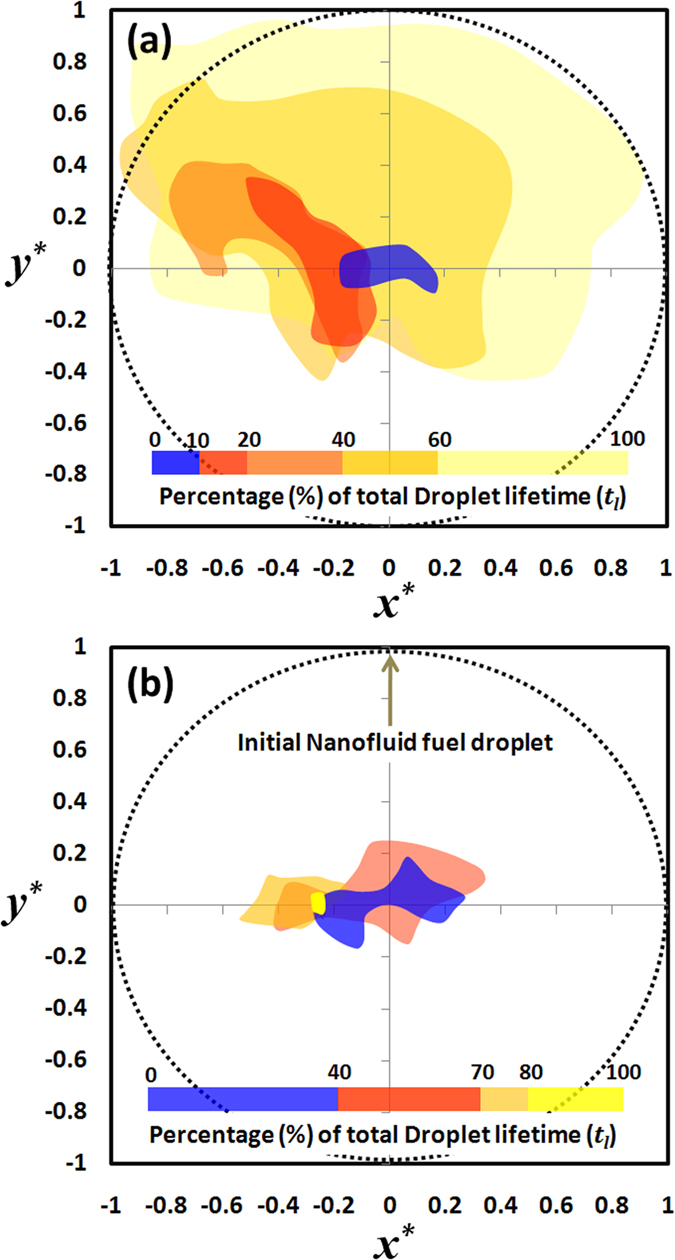
Dynamic spreading of the swept area (A) during droplet lifetime (*t*_*l*_) at (a) 0.05 wt. % initial concentration (dilute PLR) and (b) 10 wt. % (dense PLR).

**Figure 4:**
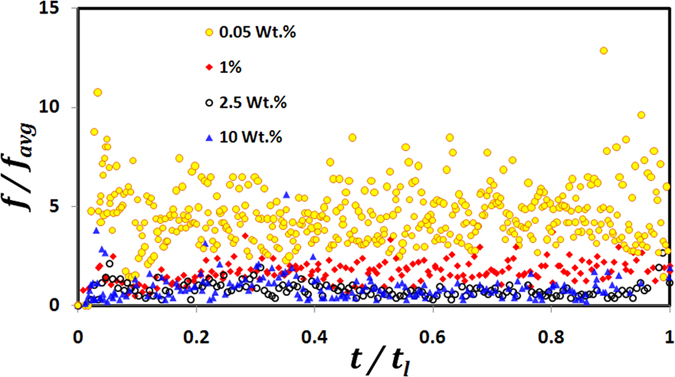
Temporal variation of dimensionless bubble ejection frequency *(f/f*_*avg*_) during droplet lifecycle. Bubble ejection frequency *f* is measured based on the ligament count and normalized with the time-mean frequency *(f*_*avg*_) for most dilute PLR (0.05 wt. %).

Insights on the transients of centroid motion can be obtained from Fig. 3 which illustrates the spreading of the swept area (*A*) with time. For brevity, comparison between dilute and dense concentrations is done based on the representative cases i.e. 10 wt. % for dense PLRs and 0.05 wt. % for dilute PLRs. Figure 3 reveals following features: (1) the swept area dilates continuously with time at 0.05 wt. % while at 10 wt. %, it exhibits a non-monotonic behavior i.e. it is constant for ~0.4*t*_*l*_ , then increases slightly for a short time period ~0.3*t*_*l*_ and then shrinks nearly to its initial value in the final stages ~0.3*t*_*l*_ and (2) the rate of spreading of *A* is clearly faster (time-averaged spreading rate of swept area is two orders of magnitude higher) and the spreading is comparatively more uniform at dilute PLRs than at dense PLRs. Uniform spreading of the swept area is indicative that the centroid motion has no directional sensitivity. This occurs because the ejection events that induce bulk displacement (i.e. centroid shifting) themselves have no preferential direction of ejection and occur continuously across the droplet surface (Fig. 1). Also, uniform ejections at dilute PLRs are aided by a markedly high *ejection frequency f* at 0.05 wt. % (Fig. 4). Here, *f* is calculated based on the *ligament count* (measured at a temporal resolution of ~3.33 ms) and is ~5–6 times higher at dilute PLRs.

**Figure 5:**
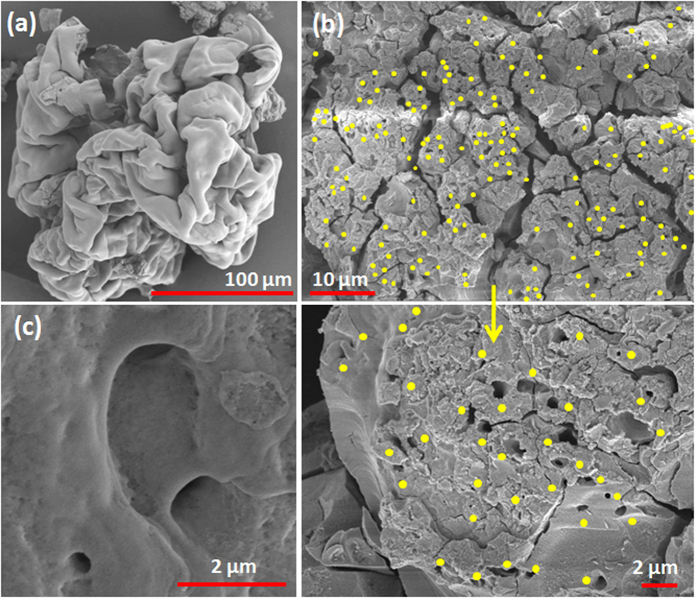
SEM micrographs of the combustion residue for a stabilized 1 wt.% nanotitania dispersion (a) overall view showing a crumpled soft shell (0.85 KX magnification), (b) magnified images of a portion of the final precipitate (4.2 KX and 9.95 KX respectively) showing uniformly distributed ejection blow-holes (yellow dot markers) and (c) magnified view of straight through ejection blow-holes with a thin-sharp peripheral edge (21.7 KX).

Starting with a low initial concentration, a higher *f* ensures that the NPs are evacuated at a faster rate from the droplet domain. This delays the crust formation process till the end and results in a weak soft shell during final stages. The shell being weak (due to depleted NP population) has equal likelihood to get punctured at any location, thereby, ensuring uniform ejections even in final stages. This is evident from the SEM micrograph of combustion residue at 1 wt. % (Fig. 5) which illustrates uniformly scattered ejection blow-holes. The degree of homogeneity in bubble ejections can be estimated by calculating a time-mean *non-uniformity parameter* 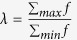. This is defined as the ratio of sum total of ejections in a quadrant with maximum *f* to sum total of ejections in a quadrant with minimum *f*. At 0.05 wt. %, *λ* is ~2.5 while at 10 wt. % it is an order higher (~18), thus, indicating that the dilute PLRs are preferable for homogenizing the injected fuel.

**Figure 6:**
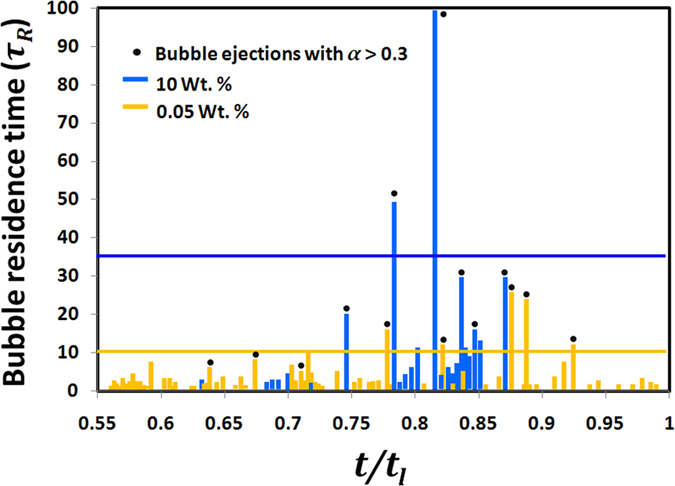
Temporal variation of bubble residence time *τ*_*R*_ *(ms)* during second-half of the droplet lifecycle. Average *τ*_*R*_ for high intensity bubble ejections with local ejection impact parameter *α*_*local,max*_ > 0.3 is shown marked for 0.05 wt.% (yellow line) and 10 wt. % (blue line).

However, at dense PLRs, the ejections and hence the efflux of NPs is suppressed (shown in Fig. 4). As a result of this increased confinement of NPs (within the droplet domain) the formation of an elastic, semi-solid crust occurs much earlier during the droplet lifetime compared to that at dilute PLRs. Moreover, rapid particle aggregation due to high surface concentration of NPs leads to the formation of a thicker and stronger crust. A stronger crust usually resists the buildup of internal pressure without undergoing rupture. This enables bubble entrapment, thereby, increasing the time delay between successive ejections. Bubble entrapment also increases the *bubble residence time* (**τ**_R_) inside the droplet (where **τ**_R_ is defined as the time elapsed since bubble inception or first observed coalescence till the onset of bubble collapse). The average **τ**_R_ for ejections with *α*_*local, max*_ > 0.3 (i.e. high intensity ejections) is ~3.5 times higher at 10 wt. % (Fig. 6). A higher **τ**_R_ promotes a rise in the bubble population, thereby, increasing the chances of bubble coalescence. This in turn promotes a rapid bubble growth with an imminent high intensity ejection that causes shell puncturing (i.e. initiation of ejection event) from a weak soft spot.

**Figure 7:**
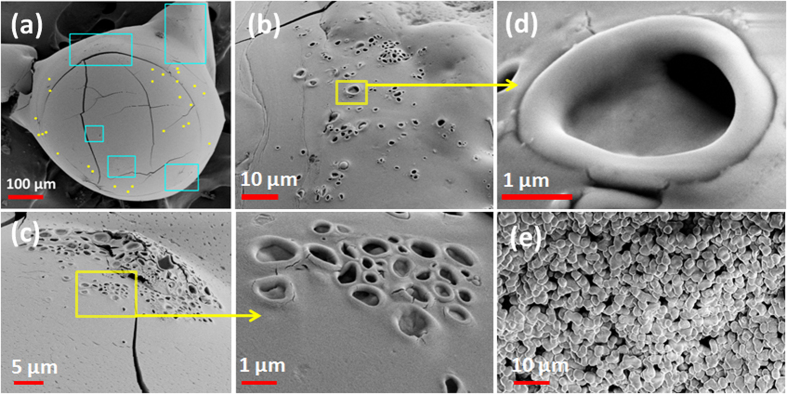
SEM photographs showing surface morphology of the combustion residue for a stabilized 10 wt. % nanotitania dispersion droplet (**a**) overall view showing a rigid precipitate structure; blue boxes indicate the non-uniformly distributed clusters of ejection blow-holes while yellow dot markers show locations of isolated blow-holes, (**b**) magnified view of a cluster, (**c**) magnified images of a group of blow-holes forming a part of a bigger cluster (Fig. 7c), (**d**) magnified view of a single ejection blow-hole with a thick peripheral edge (similar to fish mouth opening) and (**e**) magnified view of shell microstructure showing a densely packed network of fused NP aggregates.

It is noteworthy to mention here that the shell formation at dense PLRs is a complex process involving NP aggregation (through perikinetic, orthokinetic and differential settling transport mechanisms) and droplet surface regression, while it is hindered by bubble ejections; directly through shell rupture and indirectly through removal of NPs with DDs acting as parcels. Thus, shell formation is a crucial phase that controls the secondary atomization characteristics. Given the complexity, the probability of uniformly distributed weak spots across the droplet surface is quite low. Indeed these weak spots (that act as preferred ejection sites) are scattered non-uniformly (shown in Fig. 7a: SEM micrograph for a 10 wt. % case). Presence of scattered blow holes also explains why the spreading of swept area is non-uniform at dense PLRs, particularly at later stages. This entire sequential process with an intermediate bubble entrapment phase is illustrated in Fig. 1a. Clearly, this cyclic process features two competing mechanisms that are coupled: (1) gradual consolidation of the shell through increased particle aggregation (shell compaction occurs gradually since ejection events keep puncturing it intermittently) that is aided by a reduction in *f* and (2) shell rupture through high intensity ejections.

This cyclic process continues until a point where the shell is either compact enough to contain internal pressure build-up and stop ejections or the heat transfer from the enveloping flame is not sufficient (i.e. near extinction) to support bubble growth. Thus, at dense PLRs, the droplet disruptive behavior gradually shifts from continuous ejections of mild intensity (initially) to intermittent ejections of high intensity (due to bubble entrapment at later stages). Note that although high intensity ejections occur in final stages, the centroid motion nearly stagnates due to the increased droplet viscosity (as higher NP loading is retained inside the droplet) which dampens the ejection impact (suppresses the centroid motion). This restricted centroid motion also explains the observed decrease in the swept area (Fig. 3) during final stages of the droplet lifetime at dense PLRs.

**Figure 8:**
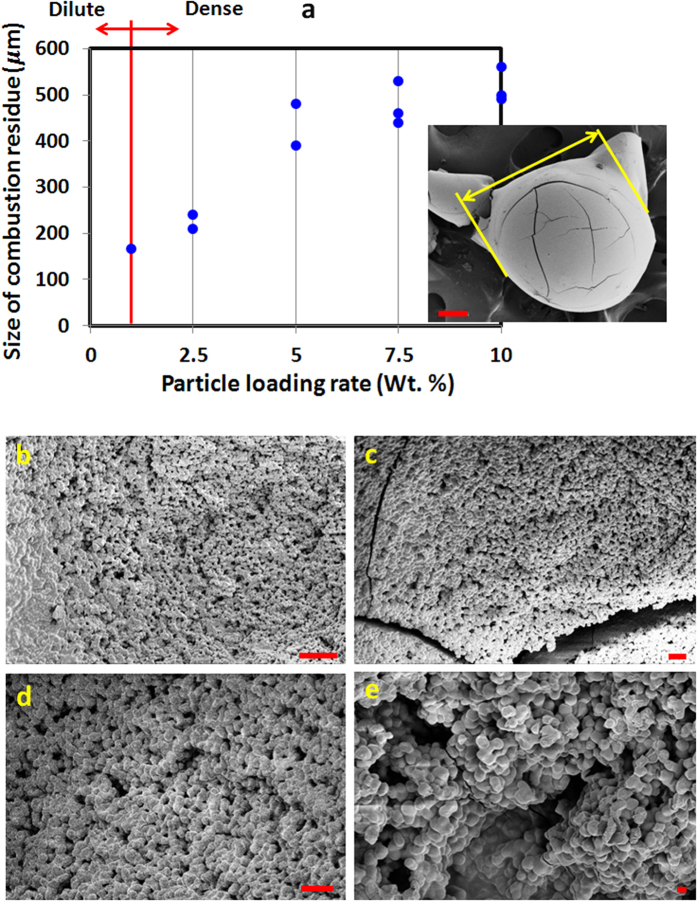
(**a**) Variation in the size of final combustion residue with initial PLR for a nanotitania dispersion droplet undergoing burning. SEM micrograph of the final precipitate at 10 wt. % is shown inset with size marked. SEM images of fused NP aggregates for (**b**) 2.5 wt. %, (**c**) 5 wt. %, (**d**) 7.5 wt. % and (**e**) 10 wt. %. Scale bar for Fig. 8b–e equals 20 μm, 2 μm, 10 μm and 2 μm respectively while the corresponding magnification is 1.45 KX, 1.82 KX, 3.63 KX and 4.85 KX respectively.

In this dynamic feedback process (where atomization modes and ejection frequency affect the crust formation and subsequently the crust modulates these modes), a critical parameter is the *instantaneous NP population* inside the droplet which determines the strength and morphology of the final combustion residue. This is crucial since the effectiveness and frequency of interparticle collisions that are key to particle aggregation (and hence the crust formation) depend directly on the number density of NPs^12^,^13^. The global shell formation process can be understood by writing the particle mass conservation equation at any time instant *t*_*n*_:
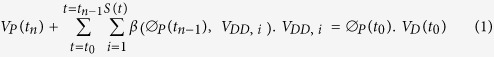


where, first term 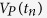
represents the volumetric NP population retained at a given instant, second term represents the NP population depletion term and third term on RHS is constant and denotes the initial NP volume (at time *t* = *t*_0_; 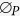: NP volume fraction and *V*_*D*_: initial droplet volume). Particularly, the rate at which NPs are evacuated from the droplet domain is controlled directly by the modes of bubble ejection (Fig. 1b) and *f*. Both these parameters cumulatively determine the size distribution of DDs and are implicit in second term through the volume of each of the DD (*V*_*DD*, *i*_) and daughter droplet population [*S*(*t*) = Ejection frequency (*f*) *Number of DDs per ejection (*n*: a function of atomization mode)]. Here, *β* is a *variable feedback or coupling factor* between first two terms in Eq. 1 and denotes the NP concentration (by Vol.) transported with each individual DD. Thus, *β* is clearly dependent on the DD size and instantaneous NP concentration. As an example, if DD size is large (through mode 4 and 5 type ejections; Fig. 1b) and *V*_*P*_(*t*_*n*_) is higher, probability of high value of *β* also increases. Since at dense PLRs, the average ejection frequency is quite low (~1.5 ejections/ms) and initial NP concentration is high (>1 wt. %), it is the first term and consequently the crust formation that dominates the secondary atomization behavior. Contrarily, at dilute PLRs, the average ejection frequency is significantly higher (>10 ejections/ms). It is postulated that for low initial concentrations (<1 wt. %), it is the second term and hence the ejections that control droplet atomization. SEM micrographs of the final combustion residue show that a compact microstructure (Fig. 7a) with a closely packed, porous network of NP aggregates (Fig. 7e) is formed at dense PLRs which remains intact (absence of surface folds) while at dilute PLRs the final precipitate shell is thin, soft and weaker (Fig. 5) that crumples due to capillary pressure^14^,^15^. Thus, with a transition in the initial PLR from dilute to dense the relative dominance shifts from high atomization frequency to NP aggregation driven shell formation which affects the size of the combustion residue. The variation in the size of final precipitate at both global and local scales as a function of initial NP loading rate is shown in the Fig. 8.

Figure 8 clearly shows that as the PLR increases the size of combustion residue also increases. The maximum residue size of ∼500 μm (i.e. half of the initial droplet radius *R*_*o*_) is observed at dense loadings (7.5 and 10 wt. %) while at 1 wt.% it is ∼150 μm (i.e. 1/10^th^ of *R*_*o*_). Below 1 wt. % (i.e. at dilute concentrations), however, there is no residue left on the wire since the high atomization frequency transports majority of the NPs from the droplet to the flame. Therefore the aggregate size for an initial loading <1 wt. % is not shown here.

Also, the surface blow holes (resulting from shell rupture during ejection events) have markedly distinct features at dense PLRs. First, they have a prominent *circumferential lip* (Fig. 7d). Second, they are present on the precipitate surface primarily in *groups* (Fig. 7c). Thirdly, these individual groups appear in *clusters* as sparsely distributed *islands* on the residue surface (Fig. 7a,b). Additionally, the blow holes are principally absent in the bottom hemisphere indicating that the bottom structure is rather rigid compared to the top-half. This is possibly due to differential settling (sedimentation) of NP aggregates in the lower half since the outflux of NPs is reduced substantially at dense PLRs. Contrarily, at dilute PLRs, the blow holes are straight through (with *no lips*) and are distributed uniformly as isolated sites across the entire residue surface (Fig. 5).

## Conclusion

Currently, significant effort is directed towards understanding the combustion behavior of nanofuel droplets with a motive of enhancing the exothermicity, energy density, ignition probability and auto ignition properties of conventional hydrocarbon fuels while reducing their soot forming potential. This is done either by varying functional properties such as particle size type and loading rate or by regulating the ambient conditions. However, the secondary break-up of nanoparticle laden droplets, characterized by the formation of ligaments and their subsequent fracture to form daughter droplets (that act as parcels for particle transport) is at pre-mature stages of investigation. In this work, we identify two key mechanisms, namely, the atomization frequency and the crust formation (through consolidation of nanoparticles) and explain how a feedback coupling between them alters the secondary break-up behavior of droplet. Key finding of the present study indicates that by varying the initial nanoparticle loading rate the relative dominance of these two mechanisms can be altered to yield microstructures with different morphologies.

In summary, although an entire spectrum of ejections with their intensity ranging from *α* ~ 0 to *α* → 1 occur at both dilute and dense PLRs^6^,^7^,^8^, their effect measured in terms of the displacement of droplet centroid is particularly subdued at dense concentrations due to increased droplet viscosity. This is a manifestation of the competition between the frequency of ejections and the semi-solid crust formation (through consolidation of NP). At dense PLRs, it is the strong shell formation (master process) that governs the secondary atomization by suppressing bubble ejections (slave process), thereby, restricting outflux of NPs. However, at dilute PLRs it is the high ejection frequency (~5–6 times; master process) that governs the secondary break-up by continuously hindering the crust formation (slave) through its continuous rupture and NP efflux.

Thus, this method of atomization control by varying initial nanoparticle concentration serves a two-fold purpose. First, homogenizing the injected fuel at dilute concentrations and second, tuning the structural morphology of the final precipitate. Since several industrial processes utilize flame synthesized powders for functional coatings, our experiments may act as a versatile platform for examining the fundamental mechanisms in burning nanofuels.

## Materials and Methods

A stabilized 3 ± 0.05 μl nanotitania dispersion droplet (Anatase TiO_2_; effective particle diameter: 4–8 nm) with a bi-component base fluid (research grade ethanol and de-ionized water) is used as a test droplet. In order to form a stable, long-term suspension with low agglomeration potential the nanosuspension is subjected to ultrasound induced cavitation for ∼30 min. Following sonication the zeta potential >35 mV (measured using zeta-PALS: Phase analysis Light Scattering analyzer from Brookhaven Instruments) for each of the loading rates. Particularly, the zeta potential is >45 ± 2 mV for a significant amount of time (∼15 min.) which is two orders of magnitude greater than the experimentation timescale (i.e droplet lifetime of ∼1–2 s). Thus, in the current experiments nanosuspensions are fairly stable with homogenously dispersed NPs and form a long-term suspension. The fuel droplet is suspended in pendant mode and combustion is achieved in normal gravity at atmospheric pressure. Atomization characteristics are investigated at seven initial PLRs ranging from 0.05, 0.5 wt. % (dilute) to 1 wt. % (intermediate) to 2.5, 5, 7.5 and 10 wt. % (dense). Dynamic viscosity (*μ*) of nanosuspensions is measured at 20 °C using a dynamic shear rheometer (DSR) for shear rates varying from 10 to 1000 s^−1^ and in this range, *μ* is nearly constant at all concentrations (variation in *μ* is within 1%).These values are detailed in Table 1 as a function of initial PLR. Other details of the experimental methodology and test facility have been described comprehensively in our previous studies^6^,^7^,^8^.

## Additional Information

**How to cite this article**: Miglani, A. and Basu, S. Coupled Mechanisms of Precipitation and Atomization in Burning Nanofluid Fuel Droplets. *Sci. Rep.* **5**, 15008; doi: 10.1038/srep15008 (2015).

## Figures and Tables

**Table 1:** Dynamic viscosity of different nanosuspensions measured at 20 °C.

**Nanosuspension (Wt. %)**	**Dynamic Viscosity (cP) at 20 °C**
Pure Ethanol (99.5%)	1.06 ± 0.022
De-ionized water	1 ± 0.002
0.05	1.05 ± 0.032
0.5	1.088 ± 0.001
1	1.145 ± 0.005
2.5	1.455 ± 0.025
5	1.83 ± 0.010
7.5	2.24 ± 0.020
10	2.7 ± 0.052
